# Alveola Dental Pyorrhoea

**Published:** 1915-07

**Authors:** 


ALVEOLA DENTAL PYORRHOEA. This is an entirely new book on a much discussed subject which attempts to explain the cause of pyorrhoea on the basis of infection by the endamoeba buccalis. The authors, Drs. Charles C. Bass and Foster M. Johns, Professors in the Medical College of Tulane University, have made exhaustive laboratory researches, and believe they have isolated the specific cause of pyorrhoea in the endamoeba and claim to have secured confirmation in successful clinical treatments of the disease. The book attempts to give in detail only the pathology and treatment from this particular view point. It is not intended as a treatise on the entire subject for dental consumption wholly, but directs attention to the medical relations and to the physician's duty in regard to it. Therefore while of great interest to the dentist, we may be disappointed in the lack of technical attention which we ordinarily have in dental literature. The dentist, however, must read it as a contribution from medical men and in medical phraseology. Personally we can not accept wholly the authors' conclusions as to therapeutic treatments, and we shall be surprised if experience bears out the predictions of the book. We shall, however, be most grateful if such result occurs. It is a timely contribution and every active worker in this field will need this book. It is published by the W. B. Saunders Co., of Philadelphia.

